# Metabolic Engineering of Probiotic *Saccharomyces boulardii* Enables Intestinal 3-Hydroxybutyrate Delivery and Alters Short-Chain Fatty Acid Profiles in Mice

**DOI:** 10.3390/foods15173006

**Published:** 2026-08-26

**Authors:** Deokyeol Jeong, Tongkewn Yoo, Jieun Woo, Luping Xu, Soo Rin Kim, Weicang Wang, Kee-Hong Kim, Eun Joong Oh

**Affiliations:** 1 Department of Food Science and Technology, Kongju National University, Yesan 32439, Republic of Korea; dyj@kongju.ac.kr; 2 Department of Food Science, Purdue University, West Lafayette, IN 47907, USA; yoo192@purdue.edu (T.Y.); woo89@purdue.edu (J.W.); xu653@purdue.edu (L.X.); wang6205@purdue.edu (W.W.); 3 Whistler Center for Carbohydrate Research, Purdue University, West Lafayette, IN 47907, USA; 4 Interdepartmental Nutrition Graduate Program, Purdue University, West Lafayette, IN 47907, USA; 5 School of Food Science and Biotechnology, Kyungpook National University, Daegu 41566, Republic of Korea; soorinkim@knu.ac.kr

**Keywords:** probiotic yeast, *Saccharomyces boulardii*, metabolic engineering, CRISPR/Cas9, 3-hydroxybutyrate, short-chain fatty acid

## Abstract

3-Hydroxybutyric acid (3-HB) is a bioactive ketone body involved in the regulation of intestinal inflammation and metabolic homeostasis. Although engineered bacterial probiotics have been developed for localized 3-HB delivery, their susceptibility to antibacterial antibiotics may limit their use during concurrent antibiotic treatment. The probiotic yeast *Saccharomyces boulardii* offers an alternative host for intestinal 3-HB delivery because of its compatibility with antibacterial antibiotics and the availability of well-established genetic engineering tools. Here, we engineered *S. boulardii* for 3-HB production using Cas9-mediated genome editing. A heterologous 3-HB biosynthetic pathway was introduced into *S. boulardii* MYA-797, and endogenous acetyl-CoA and ethanol metabolism was subsequently rewired by overexpressing *ACS1*, deleting *ADH1*, and overexpressing *ADH7*. The optimized strain, SbDY02, produced 1.7 g/L 3-HB under microaerobic conditions. Oral administration of SbDY02 to C57BL/6J mice increased fecal 3-HB and short-chain fatty acid (SCFA) concentrations by 1.89-fold and 1.68-fold, respectively, compared with mice receiving the parental strain. Repeated administration also increased fecal acetate and circulating total SCFAs, butyrate, and propionate. In human colonic epithelial cells, purified 3-HB attenuated lipopolysaccharide-induced p38 MAPK phosphorylation, supporting its direct activity toward inflammation-associated epithelial signaling. To our knowledge, this study provides the first demonstration of a 3-HB-producing probiotic yeast and links central metabolic engineering of *S. boulardii* with increased 3-HB availability, altered SCFA profiles, and a host-relevant epithelial response.

## 1. Introduction

3-Hydroxybutyrate (3-HB), also known as β-hydroxybutyrate, is a four-carbon organic acid that functions as both an energy metabolite and a signaling molecule. Although 3-HB is best known as a ketone body produced in the liver during fasting, exercise, and carbohydrate restriction, it also regulates metabolic processes, inflammatory signaling, and intestinal homeostasis [1,2,3]. These diverse biological functions have driven further interest in the physiological and therapeutic potential of 3-HB, particularly in whether its local production in the intestine can beneficially modulate the gut environment.

Advances in synthetic biology and marker-free genome editing have enabled the development of microbial systems that deliver therapeutic compounds directly to the intestine, where sustained local exposure can be difficult to achieve through conventional administration [4,5,6,7]. Bacterial species such as *Escherichia coli*, *Lactobacillus crispatus*, *Limosilactobacillus reuteri*, and *Bifidobacterium* spp. have been investigated as microbial hosts because of their safety profiles, rapid growth, and ability to survive or colonize the gastrointestinal tract [8,9,10,11,12]. For instance, oral administration of an engineered 3-HB-producing probiotic *E. coli* Nissle 1917 (EcN) strain, designated EcNL4, increased intestinal short-chain fatty acid levels, promoted the growth of beneficial bacteria, such as *Akkermansia* spp., and alleviated dextran sulfate sodium-induced colitis in mice compared with administration of the parental EcN strain [10,13]. However, the susceptibility of bacterial systems to antibiotics may limit their use during concurrent antibiotic therapy [14,15,16]. The probiotic yeast *Saccharomyces boulardii* therefore offers an attractive alternative host.

*S. boulardii* is a clinically used probiotic yeast that modulates the gut microbiota and is intrinsically compatible with antibiotic treatment [17]. It protects the intestinal environment through luminal, trophic, and mucosal mechanisms, including the neutralization of enterotoxins and the sequestration of pathogens by its cell-wall mannan and glucan matrix [17,18,19]. Beyond its role as a live probiotic, *S. boulardii* contains cell-wall oligosaccharides with prebiotic potential [20]. Compared with *Saccharomyces cerevisiae*, *S. boulardii* has a mannan-enriched cell wall, and its biomass has been shown *in vitro* to preferentially support the growth of *Bacteroides thetaiotaomicron* over *Clostridioides difficile* [20]. This selectivity was further enhanced by increasing the cell-wall mannan content [20]. *S. boulardii* is also closely related to the model yeast *S. cerevisiae*, facilitating the direct adaptation of established genome-editing tools [21,22,23]. These properties make *S. boulardii* a promising platform for the localized delivery of functional compounds such as 3-HB to the intestine. To our knowledge, *S. boulardii* has not been engineered for 3-HB production, and the effects of a 3-HB-producing probiotic yeast on intestinal and circulating short-chain fatty acid (SCFA) profiles remain unknown.

**Figure 1:**
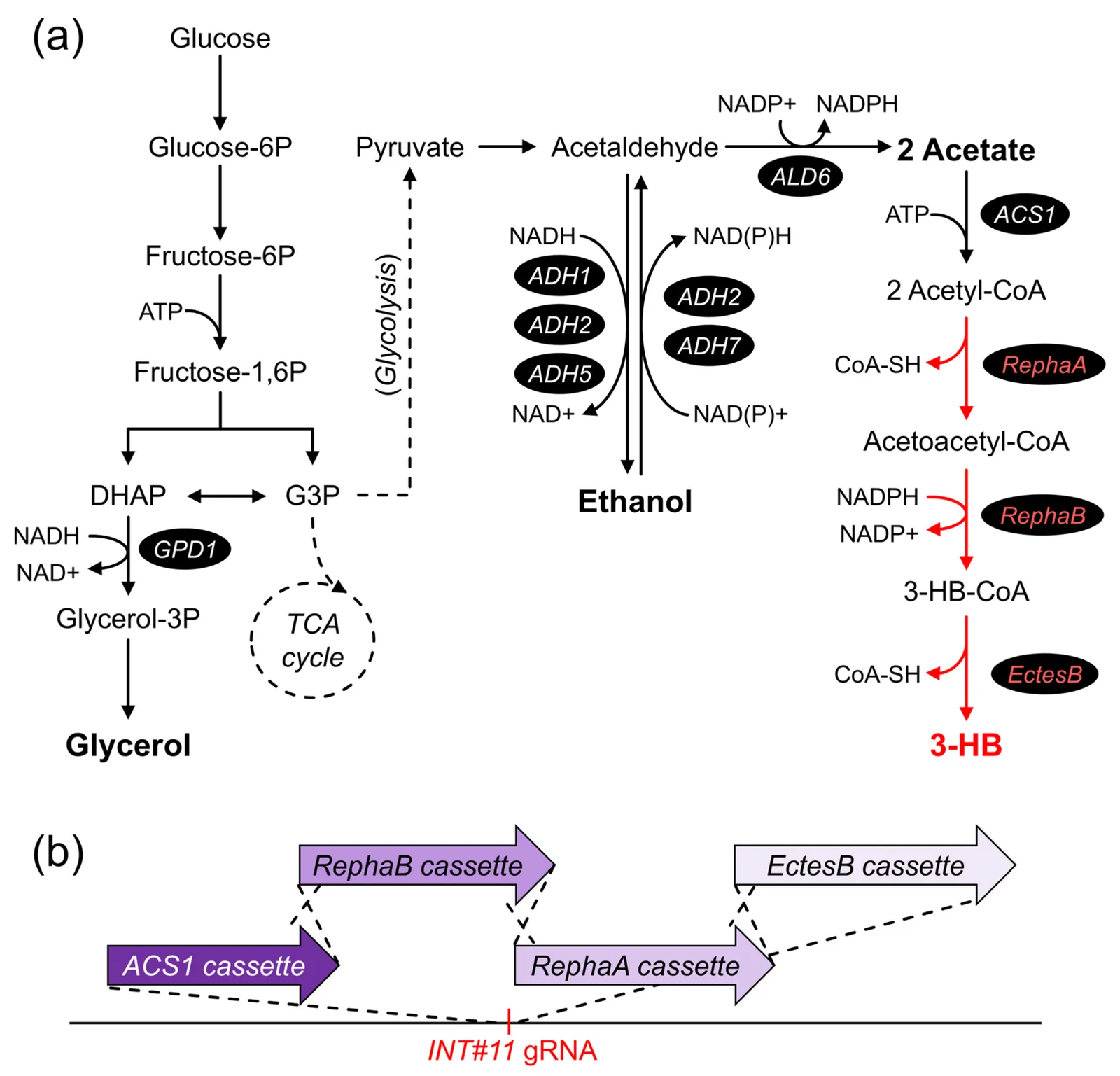
Metabolic pathway engineering and genomic integration strategy for 3-hydroxybutyrate (3-HB) production. (**a**) Schematic of endogenous carbon metabolism and the heterologous pathway introduced for 3-HB biosynthesis. Red arrows indicate the introduced reactions and directed carbon flux toward 3-HB production. *RephaA*, acetyl-CoA acetyltransferase from *Ralstonia eutropha* H16; *RephaB*, acetoacetyl-CoA reductase from *R. eutropha* H16; *EctesB*, thioesterase from *Escherichia coli*; *GPD1*, glycerol-3-phosphate dehydrogenase; *ALD6*, aldehyde dehydrogenase; *ACS1*, acetyl-CoA synthetase; *ADH1*, *ADH2*, *ADH5*, and *ADH7*, alcohol dehydrogenases; DHAP, dihydroxyacetone phosphate; G3P, glyceraldehyde 3-phosphate. (**b**) Genomic integration of the *ACS1*, *RephaA*, *RephaB*, and *EctesB* expression cassettes at intergenic region #11 (INT#11) using an INT#11-targeting single-guide RNA.

On this basis, engineering *S. boulardii* for localized metabolite production may provide an additional means of modulating the intestinal environment beyond the intrinsic probiotic and cell wall-associated properties of the parental yeast. Here, we engineered *S. boulardii* to produce 3-HB from glucose by introducing three heterologous biosynthetic genes, overexpressing *ACS1* to increase acetyl-CoA availability, and modifying the expression of alcohol dehydrogenase genes (Figure 1a). We then evaluated whether the resulting strain altered intestinal and circulating SCFA profiles compared with the parental probiotic strain. The optimized strain, SbDY02, produced 1.7 g/L 3-HB under the tested cultivation conditions. Oral administration of SbDY02 increased fecal 3-HB and altered intestinal and circulating short-chain fatty acid profiles in mice, while 3-HB attenuated lipopolysaccharide-induced p38 mitogen-activated protein kinase (MAPK) activation in human intestinal epithelial cells. These findings establish engineered *S. boulardii* as a yeast-based platform for localized metabolite delivery and modulation of the intestinal metabolic environment.

## 2. Materials and Methods

### 2.1. Strains, Plasmids, and Culture Conditions

The *S. boulardii* strains and plasmids used in this study are listed in Table 1. *S. boulardii* MYA-797 was used as the parental strain for 3-hydroxybutyric acid (3-HB) production. Yeast strains were precultured in yeast extract-peptone (YP) medium (10 g/L yeast extract and 20 g/L peptone) supplemented with 20 g/L glucose (YPD20) at 30 °C and 250 rpm. For 3-HB production, precultures were inoculated into 25 mL of YP medium containing 40 g/L glucose (YPD40) in 125 mL Erlenmeyer flasks at an initial optical density at 600 nm (OD_600_) of 1.0. Cultures were incubated at 30 °C and 100 rpm. *E. coli* TOP10 (Invitrogen, Carlsbad, CA, USA) was used for the propagation and maintenance of single-guide RNA (sgRNA) plasmids. *E. coli* cells were cultured in Luria–Bertani medium (5 g/L yeast extract, 10 g/L tryptone, and 10 g/L NaCl) supplemented with 100 μg/mL ampicillin (LBA) at 37 °C for 12 h, and plasmids were extracted using the QIAprep Spin Miniprep Kit (Qiagen, Germantown, MD, USA) according to the manufacturer’s instructions.

### 2.2. Construction of 3-Hydroxybutyric Acid-Producing Strain

Gene integration and deletion in *S. boulardii* were performed using a CRISPR/Cas9 system as previously described [23]. The pRS41K-Cas9 plasmid was used for Cas9 expression [24] and pRS42H-INT#11 was used for sgRNA expression targeting intergenic region 11 (INT#11) [25]. Donor DNA fragments were PCR-amplified using Q5 High-Fidelity 2× Master Mix (New England Biolabs, M0492, Ipswich, MA, USA) with the primers listed in Table S1. To construct the 3-HB biosynthetic pathway, codon-optimized *RephaA* (Accession number PZ713508) and *RephaB* (Accession number PZ713509) were synthesized by Twist Bioscience (San Francisco, CA, USA). *EctesB* (Accession number CP184745.1) was PCR-amplified from the genome of *E. coli* TOP10, and *ACS1* was PCR-amplified from the genome of *S. boulardii* MYA-797 (Table S2). The four expression cassettes (*PGK1*_P_*-ACS1-ADH7*_T_, *CCW12*_P_*-RephaB-CYC1*_T_, *TDH3*_P_*-RephaA-ADH4*_T_, and *FBA1*_P_*-EctesB-MDH1*_T_) were integrated at *INT#11* in the *S. boulardii* genome (Figure 1b) [26].

To reduce ethanol accumulation, sgRNAs targeting the alcohol dehydrogenase genes (*ADH1*, *ADH2*, *ADH5*, and *ADH7*) were designed using CHOPCHOP (https://chopchop.cbu.uib.no, (accessed on 10 April 2023)) and cloned into pRS42H using the fast cloning method [27]. The 100 bp donor DNA fragments used for gene deletion were generated by PCR using complementary overlapping oligonucleotides without a DNA template. For *ADH7* overexpression, an additional donor fragment was constructed to replace the native *ADH7* promoter with the strong constitutive *TDH3* promoter. Yeast transformations were performed using the lithium acetate method [28]. Transformants were selected on YPD20 agar supplemented with 300 μg/mL G418 sulfate and 300 μg/mL hygromycin B. Correct genome editing was verified by colony PCR using OneTaq DNA Master Mix (New England Biolabs, M0482) with the primers listed in Table S1.

### 2.3. Measurement of Cell Growth and Extracellular Metabolites

Yeast cell growth was monitored by measuring the optical density at 600 nm (OD_600_) using a BioMate 160 UV-Vis spectrophotometer (Thermo Scientific, Waltham, MA, USA). Dry cell weight (DCW) was estimated using a conversion factor of 0.5 g DCW/L per OD_600_ unit. Extracellular metabolites (glucose, glycerol, acetate, 3-HB, and ethanol) in culture supernatants were quantified by high-performance liquid chromatography (HPLC; Agilent Technologies, 1260 series, Santa Clara, CA, USA) equipped with a refractive index (RI) detector. Chromatographic separation was achieved using a Rezex-ROA Organic Acid H^+^ (8%) column (150 mm × 4.6 mm; Phenomenex Inc., Torrance, CA, USA) maintained at 65 °C. The mobile phase was 5 mM H_2_SO_4_ delivered at a constant flow rate of 0.6 mL/min.

### 2.4. Animals and Experimental Design

Male C57BL/6J mice aged 25 weeks were obtained from the Jackson Laboratory (Bar Harbor, ME, USA). After one week of acclimation, the mice were maintained under a 12 h light/dark cycle with ad libitum access to standard chow and water throughout the experiment. Mice were randomly assigned to receive phosphate buffered saline (PBS) alone (n = 5), wild-type yeast *S. boulardii* MYA-797 (WT Sb; n = 5), or the engineered 3-HB-producing strain (SbDY02; n = 6). Yeast cells were orally administered at a dose of 1 × 10^9^ cells suspended in 150 μL PBS. Fecal samples were collected at the indicated time points and stored at −80 °C until analysis. After 2 or 14 days of the experiment, blood samples were collected into heparinized tubes by submental bleeding and centrifuged at 1000× *g* for 15 min to isolate plasma. Plasma samples were stored at −80 °C until analysis.

### 2.5. Fecal and Plasma Metabolite Analysis Using LC/QQQ

An Agilent 1200 Rapid Resolution liquid chromatography (LC) system coupled to an Agilent 6460 series QQQ mass spectrometer (MS) was used to analyze 3-HB, acetic acid, propionic acid, and butyric acid in each sample. A Waters Atlantis T3 (2.1 mm × 75 mm, 3 um) column (Waters Corporation Milford, MA, USA) was used for LC separation. HPLC grade water with 0.1% formic acid (*v*/*v*) was used as mobile phase A. HPLC grade acetonitrile with 0.1% formic acid (*v*/*v*) was used as mobile phase B. The linear LC gradient was as follows: time 0 min, 10% B; time 0.5 min, 10% B; time 12 min, 40% B; time 13 min, 98% B; time 14 min, 10% B; time 20 min, 10% B. The flow rate was 0.4 mL/min. Multiple reaction monitoring (MRM) was used for MS analysis. The data were acquired in positive electrospray ionization (ESI) mode. The jet stream ESI interface had a gas temperature of 325 °C, gas flow rate of 10 L/min, nebulizer pressure of 40 psi, sheath gas temperature of 250 °C, sheath gas flow rate of 7 L/min, capillary voltage of 4000 V in positive mode, and nozzle voltage of 1500 V. The ΔEMV was 200. The concentrations of 3-HB and the other SCFAs were determined from the relative response between aniline-labeled sample versus ^13^C_6_ aniline-labeled standard [29]. All data were analyzed with Agilent MassHunter Quantitative Analysis (Version 10.1).

### 2.6. Cell Culture and Treatment

Human colonic epithelial HCT-8 cells (ATCC, Manassas, VA, USA) were cultured in Roswell Park Memorial Institute (RPMI)-1640 medium with L-glutamine (10-040-CV, Corning Inc., Corning, NY, USA) containing 10% fetal bovine serum (FBS) and maintained at 37 °C in a humidified atmosphere with 5% CO_2_. For Western blot analysis, cells were serum-starved for 8 h in RPMI medium supplemented with 1% FBS. Cells were then pretreated with 3-HB (166898, Sigma-Aldrich, St. Louis, MO, USA; 10 or 100 μM in PBS) for 30 min, followed by stimulation with lipopolysaccharide (LPS; 1 μg/mL, Sigma-Aldrich, St. Louis, MO, USA) for 30 min. For qPCR analysis, cells were serum-starved in RPMI medium, pretreated with 3-HB (100 μM in PBS) for 1 h, and then stimulated with LPS (10 ng/mL) for 2 h. Untreated cells and cells treated with LPS alone were included as controls.

### 2.7. Western Blot Analysis

Following treatment, HCT-8 cells were lysed in RIPA buffer (Boston Bioproducts, BP-115DG, Milford, MA, USA) supplemented with a protease inhibitor cocktail (Thermo Fisher Scientific, Waltham, MA, USA, Cat# A32963). Protein samples were separated by 10% SDS-PAGE and transferred to nitrocellulose membranes. Membranes were blocked with EveryBlot blocking buffer (Bio-Rad, Hercules, CA, USA, Cat# 12010020) and incubated overnight at 4 °C with primary antibodies against phosphorylated p38 MAPK (Cell Signaling Technology, Danvers, MA, USA, Cat# 9211), total p38 MAPK (Cell Signaling Technology, Cat# 9212), and β-actin (Cell Signaling Technology, Cat# 3700). Membranes were subsequently incubated with fluorophore-conjugated secondary antibodies, IRDye^®^ 800CW (LI-COR Biosciences, Lincoln, NE, USA, Cat# 926-32211) or IRDye^®^ 680RD (LI-COR Biosciences, Cat# 926-68180). Fluorescence signals were detected using the LI-COR Odyssey LX imaging system, and band intensities were quantified using ImageJ (version 1.54g). β-actin was used as a loading control, and phosphorylated p38 MAPK abundance was normalized to total p38 MAPK.

### 2.8. RNA Extraction and Quantitative Real-Time PCR (RT-qPCR)

Total RNA was extracted with TRIzol reagent (Invitrogen, Carlsbad, CA, USA; #15596018) according to the manufacturer’s protocol and reverse transcribed using the High-Capacity cDNA Reverse Transcription Kit (Applied Biosystems, Waltham, MA, USA; #4368814). RT-qPCR was performed using PowerUp SYBR Master Mix (Thermo Fisher Scientific) on a CFX96 Real-Time PCR Detection System (Bio-Rad, Hercules, CA, USA). The cycling conditions were 95 °C for 2 min, followed by 40 cycles of 95 °C for 2 s and 60 °C for 30 s. Relative expression was calculated using the 2^−ΔΔCt^ method, with *GAPDH* as the reference gene. Primer sequences are listed in Table S3.

### 2.9. Statistical Analysis

Data are presented as the mean ± standard deviation (s.d.). Data processing was performed using Microsoft Excel 365 (Microsoft Corp, Redmond, WA, USA), and statistical analyses were conducted using IBM SPSS Statistics (version 25, Armonk, NY, USA). Statistical significance was determined using either Student’s *t*-test or one-way ANOVA followed by Tukey’s HSD test with *p* < 0.05 considered statistically significant. Fermentation and Western blot experiments were performed in triplicate repeats, and mouse experiments included five mice each in the PBS and wild-type *S. boulardii* MYA-797 strain (WT Sb) groups and six mice in the engineered 3-HB-producing strain (SbDY02) group.

## 3. Results and Discussion

### 3.1. Engineering S. boulardii for 3-HB Production

**Figure 2:**
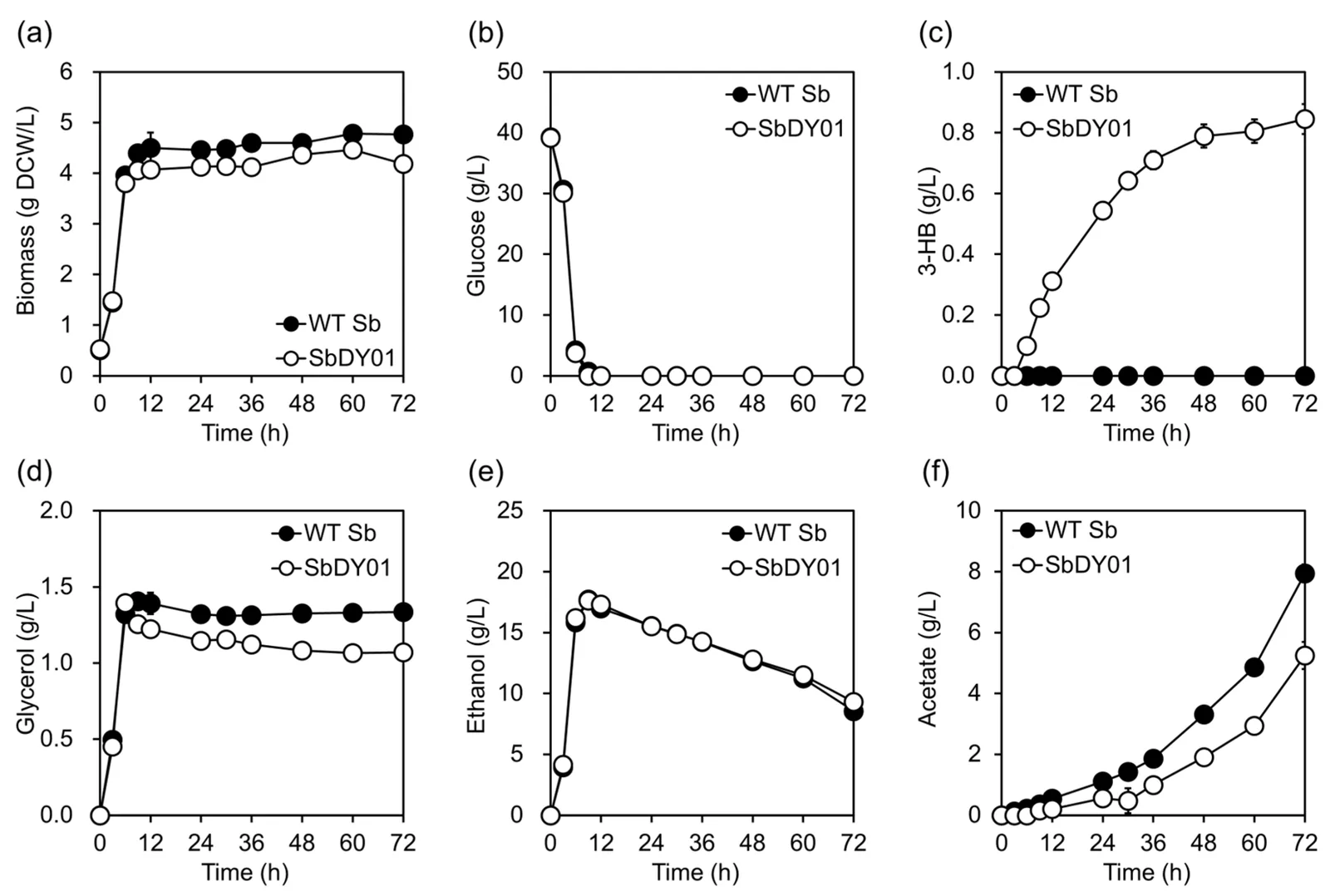
Fermentation profiles of wild-type *S. boulardii* and the engineered 3-HB-producing strain SbDY01. Wild-type *S. boulardii* (WT Sb) and SbDY01 were cultured in YP medium containing 40 g/L glucose at 30 °C and shaken at 100 rpm, with an initial biomass concentration of 0.5 g DCW/L. (**a**) Biomass formation, (**b**) glucose consumption, and (**c**–**f**) metabolite production ((**c**) 3-HB; (**d**) glycerol; (**e**) ethanol; (**f**) acetate) were compared. Data are presented as the mean ± s.d. of three biological replicates.

To establish 3-HB production in *S. boulardii*, three codon-optimized heterologous genes encoding the 3-HB biosynthetic pathway (*RephaA*, *RephaB*, and *EctesB*) were integrated into the genome of the parental strain MYA-797. The endogenous acetyl-CoA synthetase gene (*ACS1*), which converts acetate to acetyl-CoA, was also overexpressed to increase precursor availability. The resulting strain was designated SbDY01 (Figure 1). To evaluate 3-HB production from glucose, SbDY01 was cultured with glucose as the sole carbon source and compared with the wild-type strain (Figure 2). The wild-type strain completely consumed 40 g/L glucose within 9 h and accumulated 7.9 g/L acetate over 72 h but did not produce detectable 3-HB (Figure 2). In contrast, SbDY01 accumulated 5.2 g/L acetate and produced 0.8 g/L 3-HB (Figure 2c,f). The reduction in acetate accumulation, together with 3-HB production, was consistent with increased conversion of acetate to acetyl-CoA and subsequent flux through the heterologous 3-HB pathway. SbDY01 also showed a 12.5% reduction in biomass formation compared with the wild-type strain (Figure 2a), consistent with previous reports of impaired growth in 3-HB-producing *S. cerevisiae* strains [30,31]. This growth reduction may reflect competition for acetyl-CoA between biomass formation and 3-HB biosynthesis. Although these results demonstrate the feasibility of producing 3-HB in probiotic *S. boulardii*, substantial ethanol accumulation indicated that the native ethanol pathway remained a major competing route that diverted carbon away from 3-HB production.

### 3.2. Modulation of Alcohol Dehydrogenase Expression Enhances 3-HB Production

**Figure 3:**
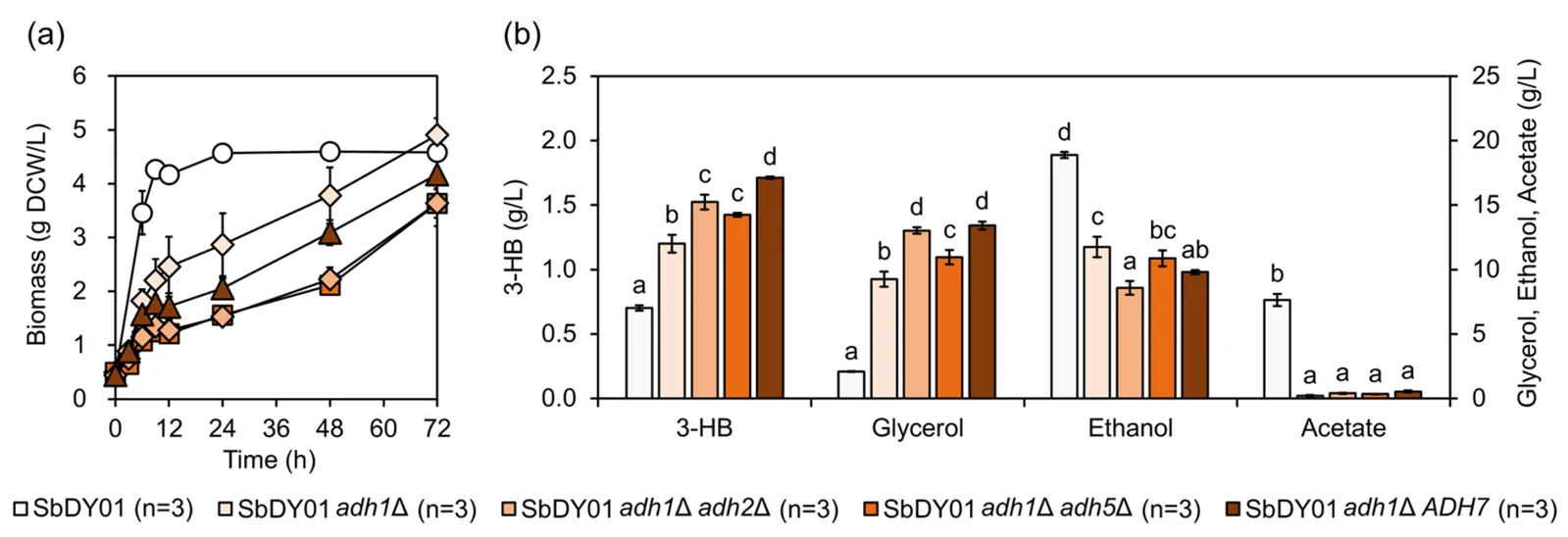
Effects of alcohol dehydrogenase gene modulation on biomass formation and metabolite production in SbDY01. Strains were cultured in YP medium containing 40 g/L glucose at 30 °C and shaken at 100 rpm, with an initial biomass concentration of 0.5 g DCW/L. (**a**) Biomass formation over 72 h. (**b**) Concentrations of 3-HB, glycerol, ethanol, and acetate after 72 h of cultivation. Data are presented as the mean ± s.d. of three biological replicates. Different letters (a–d) indicate statistically significant differences (Tukey’s HSD tests, *p* < 0.05).

We hypothesized that reducing flux through the competing ethanol pathway would increase precursor availability for 3-HB biosynthesis. First, *ADH1*, which encodes the major alcohol dehydrogenase responsible for the reduction of acetaldehyde to ethanol, was deleted [32]. The *ADH1* deletion reduced ethanol accumulation by 37.7%, to 11.8 g/L, and increased the 3-HB titer to 1.2 g/L (Figure 3). Acetate accumulation significantly decreased to 0.2 g/L, whereas glycerol production increased approximately 4.4-fold, to 9.2 g/L, relative to SbDY01. This increase in glycerol production probably reflects its role as an alternative redox sink that facilitates NADH reoxidation following disruption of ethanol formation [33,34]. Additional deletion of the auxiliary alcohol dehydrogenase genes *ADH2* or *ADH5* increased 3-HB production to 1.5 and 1.4 g/L, respectively (Figure 3). However, these modifications further increased glycerol accumulation and markedly impaired biomass formation. Such growth defects could compromise strain fitness and persistence in the gastrointestinal tract, limiting its effectiveness as a live biotherapeutic platform [35,36,37].

To improve metabolic performance while maintaining cell growth, we next overexpressed *ADH7* to promote ethanol recycling. *ADH7* catalyzes the reverse conversion of ethanol to acetaldehyde. *ADH7* overexpression partially restored biomass formation and reduced ethanol accumulation to 9.8 g/L by promoting the conversion of ethanol to acetaldehyde (Figure 3b). The resulting strain produced 1.7 g/L 3-HB, the highest titer obtained in this study. Although direct titer comparisons across probiotic hosts are limited by differences in cultivation conditions, the 1.7 g/L titer demonstrates that substantial 3-HB production can be achieved through central metabolic rewiring of *S. boulardii* [10]. Glycerol accumulation nevertheless increased to 13.4 g/L, indicating that redox imbalance remained a major constraint on 3-HB production (Figure 3b).

Collectively, these results show that modulating the alcohol dehydrogenase expression significantly increased 3-HB production but also promoted glycerol formation as a compensatory response to altered redox metabolism. Further improvements may require coordinated attenuation of glycerol biosynthesis, increased conversion of acetaldehyde to acetate, and optimization of intracellular cofactor balance. The SbDY01 derivative carrying *ADH1* deletion and *ADH7* overexpression was designated as SbDY02 and used in subsequent mouse studies.

### 3.3. Oral Administration of SbDY02 Alters Intestinal and Circulating Metabolite Profiles in Mice

**Figure 4:**
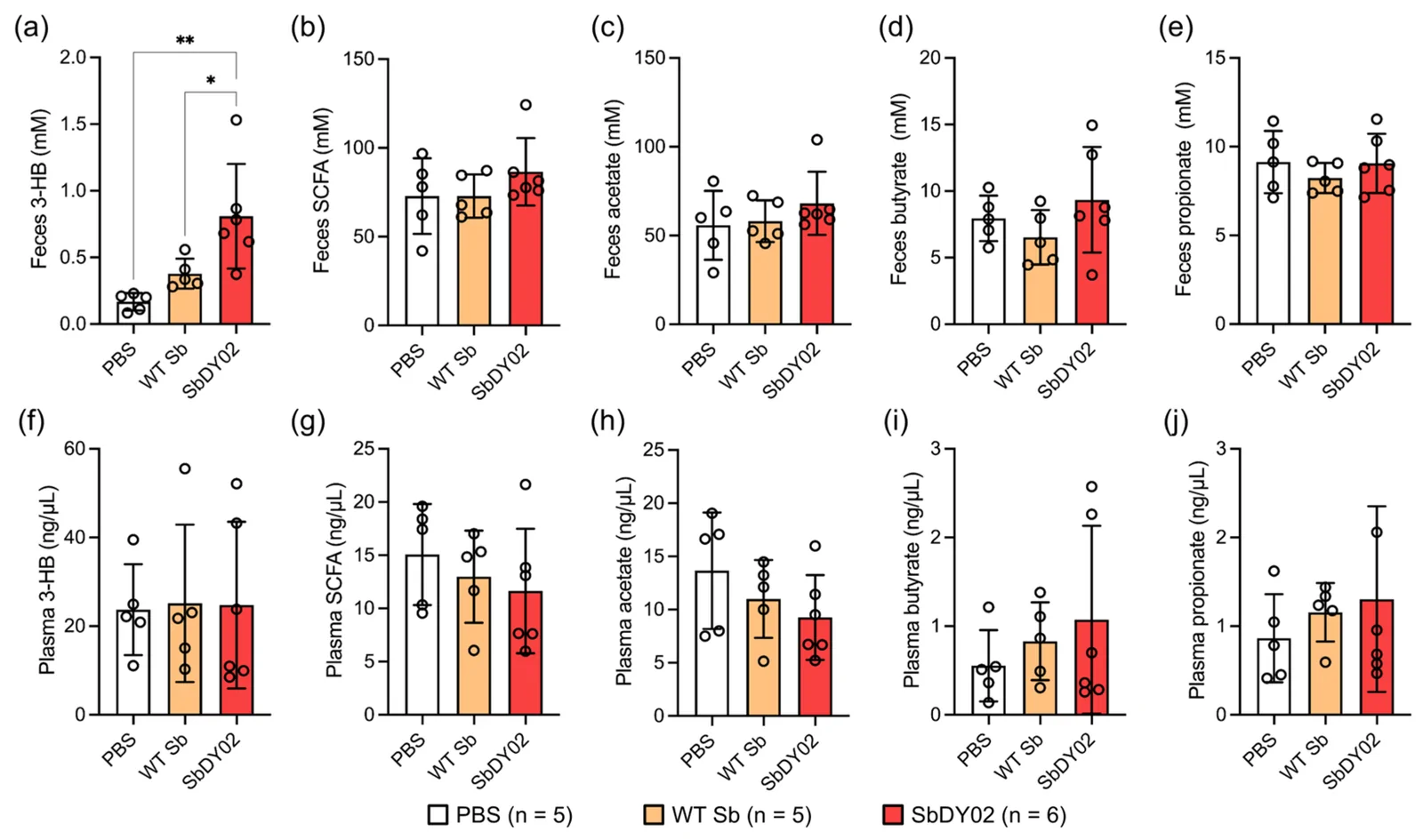
Effects of short-term SbDY02 administration on fecal and plasma 3-HB and SCFA profiles. Fecal and plasma samples were collected 48 h after oral administration of phosphate-buffered saline (PBS) vehicle (n = 5), wild-type *S. boulardii* MYA-797 (WT Sb; n = 5), or the engineered 3-HB-producing *S. boulardii* strain (SbDY02; n = 6). (**a**–**e**) Fecal concentrations of (**a**) 3-HB, (**b**) total SCFAs, (**c**) acetate, (**d**) butyrate, and (**e**) propionate. (**f**–**j**) Plasma concentrations of (**f**) 3-HB, (**g**) total SCFAs, (**h**) acetate, (**i**) butyrate, and (**j**) propionate. Data are presented as the mean ± s.d. Asterisks indicate statistically significant differences (Student’s *t*-test, * *p* < 0.05 and ** *p* < 0.01).

To assess the early effects of oral SbDY02 administration on intestinal 3-HB and SCFA profiles, fecal and plasma metabolites were measured 48 h after treatment (Figure 4). Fecal 3-HB concentrations were significantly higher in SbDY02-treated mice than in mice receiving PBS or wild-type *S. boulardii* (WT Sb), indicating increased intestinal 3-HB availability (Figure 4a). In contrast, fecal concentrations of total SCFAs, acetate, butyrate, and propionate did not differ significantly among the groups (Figure 4a–e). Plasma concentrations of 3-HB and individual or total SCFAs also remained unchanged at this time point (Figure 4f–j). Thus, short-term SbDY02 administration increased fecal 3-HB without producing detectable changes in circulating 3-HB or SCFA concentrations.

**Figure 5:**
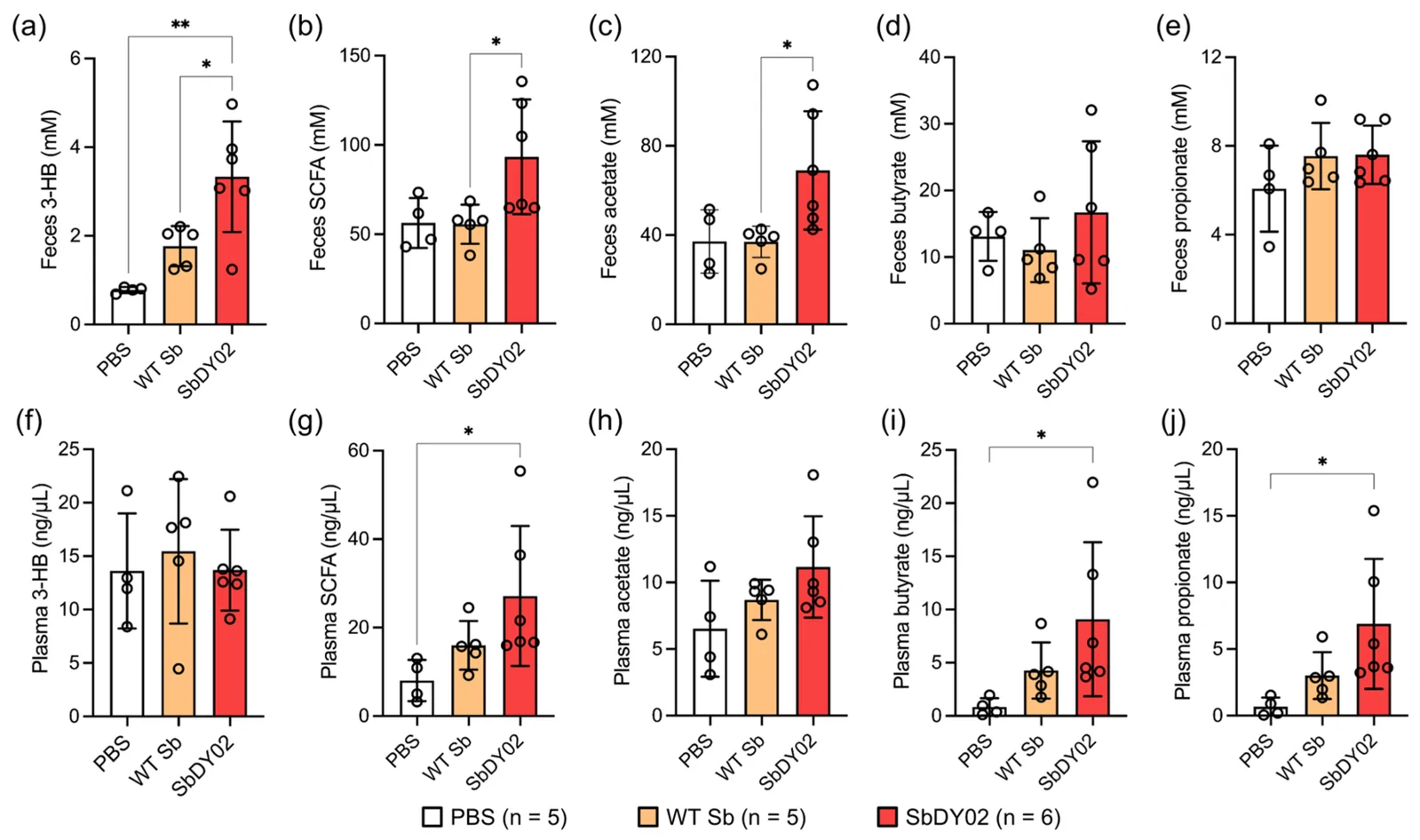
Effects of daily SbDY02 administration for 14 days on fecal and plasma 3-HB and SCFA profiles. Fecal and plasma samples were collected 14 days after oral administration of phosphate-buffered saline (PBS) vehicle (n = 5), wild-type *S. boulardii* MYA-797 (WT Sb; n = 5), or the engineered 3-HB-producing *S. boulardii* strain (SbDY02; n = 6). (**a**–**e**) Fecal concentrations of (**a**) 3-HB, (**b**) total SCFAs, (**c**) acetate, (**d**) butyrate, and (**e**) propionate. (**f**–**j**) Plasma concentrations of (**f**) 3-HB, (**g**) total SCFAs, (**h**) acetate, (**i**) butyrate, and (**j**) propionate after 14 days of daily oral administration. Data are presented as the mean ± s.d. Asterisks indicate statistically significant differences (Student’s *t*-test, * *p* < 0.05 and ** *p* < 0.01).

We next examined whether repeated administration produced more pronounced changes in intestinal and circulating metabolite profiles. Mice received PBS, WT Sb or SbDY02 once daily for 14 days, after which fecal and plasma metabolites were measured. Fecal 3-HB concentrations were higher in SbDY02-treated mice than in the PBS- and WT Sb-treated groups (Figure 5a), indicating that repeated administration sustained increased 3-HB availability in the intestinal lumen.

Total fecal SCFA concentrations showed a moderate increase in SbDY02-treated mice on day 14 (Figure 5b). Among the individual SCFAs, acetate was significantly increased, whereas butyrate and propionate did not differ significantly among the groups (Figure 5c–e). Because acetate was the most abundant fecal SCFA, its increase probably accounted for much of the change in total fecal SCFAs. These results indicate that repeated SbDY02 administration was associated with changes in the fecal SCFA profile. Consistent with these findings, the engineered 3-HB-producing probiotic *E. coli* EcNL4 was previously shown to increase fecal 3-HB and SCFA levels in mice [10]. In addition, EcNL4 promoted the expansion of beneficial bacteria, including *Akkermansia* spp., and protected against dextran sulfate sodium (DSS)-induced colitis [10]. Notably, *Akkermansia* spp. have been reported to naturally produce 3-HB [13]. However, because gut microbial composition and metabolic activity were not measured in the present study, it remains unclear whether SbDY02 directly altered SCFA-producing microbial populations or indirectly affected SCFA metabolism.

Plasma 3-HB concentrations did not differ significantly among the groups on day 14, suggesting that the increase in fecal 3-HB remained largely confined to the intestinal compartment (Figure 5f). By contrast, total plasma SCFA concentrations were significantly higher in SbDY02-treated mice than in PBS-treated mice (Figure 5g). Plasma butyrate and propionate concentrations were also significantly increased, whereas acetate showed a nonsignificant upward trend (Figure 5h–j). Notably, the plasma SCFA profile differed from that observed in feces. Although fecal butyrate and propionate concentrations were unchanged, their circulating concentrations increased following SbDY02 administration. The difference may reflect the distinct processes represented by fecal and plasma measurements. Fecal SCFAs comprise the fraction remaining after intestinal absorption and utilization and therefore do not directly reflect total microbial production [38]. Increased circulating butyrate and propionate may consequently result from enhanced intestinal production or absorption, altered host utilization, or metabolic changes that are not captured by fecal measurements alone.

Together, these findings indicate that repeated SbDY02 administration increased intestinal 3-HB availability and was associated with altered fecal and circulating SCFA profiles, including increased fecal acetate and elevated plasma butyrate and propionate. These changes may be physiologically relevant because acetate, propionate, and butyrate contribute to intestinal barrier function, mucosal immune regulation, and host metabolic homeostasis [39,40]. However, the observed changes in SCFA profiles cannot be attributed solely to an increase in intestinal 3-HB production. The parental *S. boulardii* strain itself may also affect the gut microbiota and intestinal metabolism through changes in microbial community structure and metabolite profiles [41]. In addition, the concentrations of 3-HB accumulated in the feces and plasma of SbDY02-treated mice were significantly lower than the tolerability limits and toxicity thresholds reported in previous *in vivo* studies, suggesting a low probability of acute toxicity [42,43]. Nevertheless, the absence of a formal biosafety analysis in this study remains a limitation. Therefore, future studies will be required not only to conduct extensive longer-term safety evaluations but also to examine gut microbial composition, intestinal SCFA transport, epithelial barrier function, and inflammatory responses to define the mechanisms through which SbDY02 alters intestinal and systemic metabolite profiles.

### 3.4. 3-HB Attenuates LPS-Induced p38 MAPK Activation in Human Colonic Epithelial Cells

**Figure 6:**
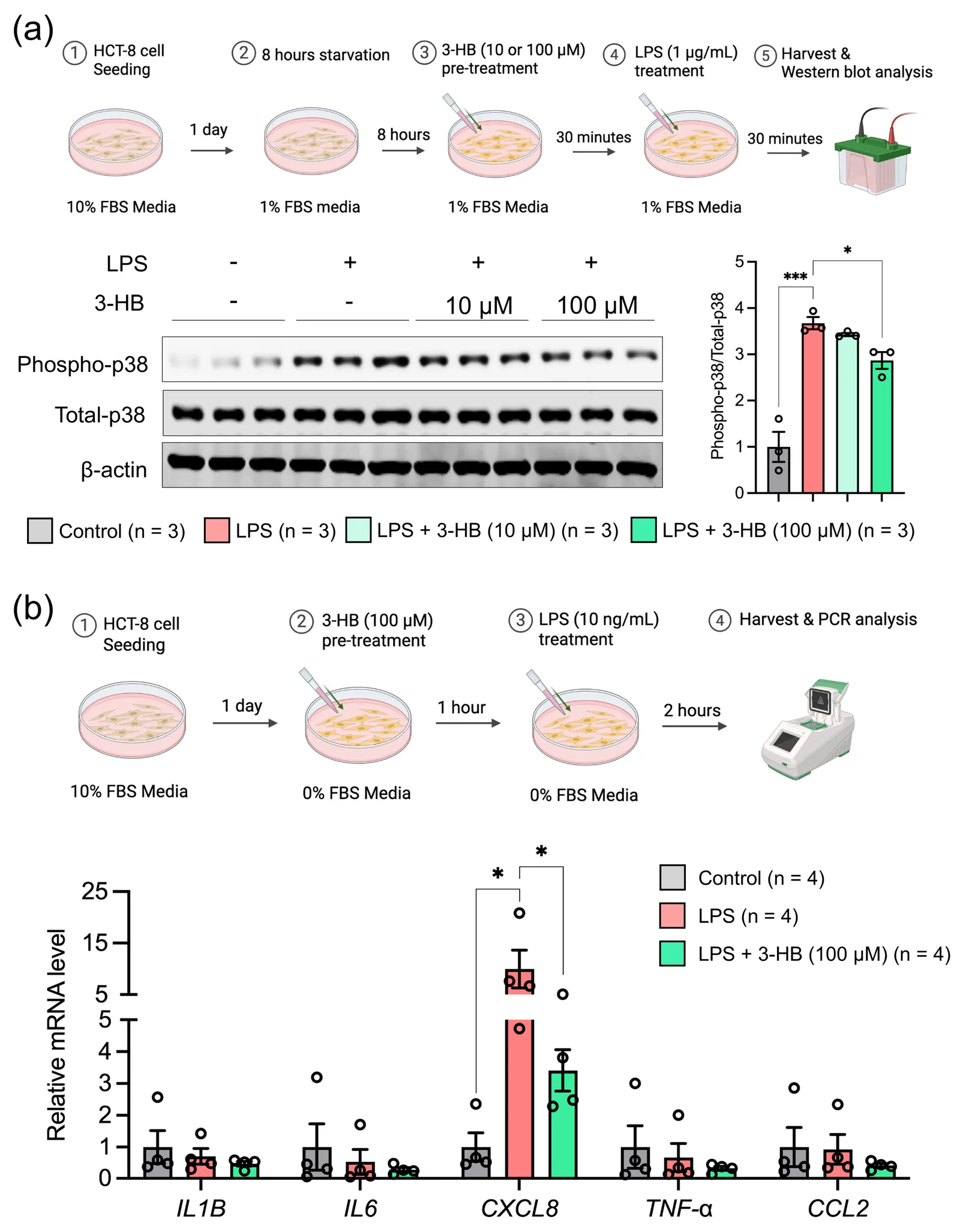
3-HB attenuates LPS-induced p38 MAPK activation and *CXCL8* expression in HCT-8 human colonic epithelial cells. Schematics of the *in vitro* experimental designs are shown for each assay. (**a**) HCT-8 cells were pretreated with 3-HB (10 or 100 μM) for 30 min and then stimulated with lipopolysaccharide (LPS, 1 μg/mL) for 30 min. Phosphorylated p38 (phospho-p38), total p38 MAPK, and β-actin were analyzed by Western blotting. Representative immunoblots and densitometric quantification of the phospho-p38/total p38 ratio are shown. β-actin was used as a loading control. (**b**) HCT-8 cells were pretreated with 3-HB (100 μM) for 1 h and then stimulated with LPS (10 ng/mL) for 2 h. *CXCL8* mRNA expression was measured by RT-qPCR. Data are presented as the mean ± s.d. (n = 3 independent experiments for phospho-p38; n = 4 for *CXCL8*). Statistical significance was determined by one-way ANOVA (phospho-p38) or the Kruskal–Wallis test (*CXCL8*), followed by the Student–Newman–Keuls post hoc test. (* *p* < 0.05, *** *p* < 0.001). Illustration created by BioRender.com.

Given that mouse experiments demonstrated increased intestinal 3-HB availability but could not distinguish the direct effects of 3-HB from those of the probiotic yeast, we next examined the response of human colonic epithelial cells to 3-HB. The concentrations used *in vitro* (10 and 100 μM, respectively) were lower than the fecal concentrations measured in SbDY02-treated mice, which were approximately 0.8 mM at 48 h and 3.3 mM at day 14, and were within the same order of magnitude as the plasma concentrations detected in the mice. Thus, these concentrations were used to test the direct biological activity of 3-HB at conservative exposures rather than to quantitatively reproduce the intestinal lumen. Although fecal concentrations do not directly reflect epithelial-surface exposure and plasma 3-HB was not significantly increased by SbDY02, the observed concentrations support biological plausibility of the *in vitro* treatment range. Moreover, this localized and continuous release of 3-HB by SbDY02 provides sufficient physiological exposure to significantly alter the luminal metabolic environment, as evidenced by the *in vivo* SCFA shifts. Given the role of p38 MAPK signaling in intestinal pro-inflammatory responses [44], we examined whether 3-HB modulates this pathway in colonic epithelial cells. LPS treatment increased p38 phosphorylation compared with untreated controls (Figure 6a). Notably, pretreatment with 100 μM 3-HB attenuated the LPS-induced increase in the phospho-p38/total-p38 ratio (Figure 6a). Consistently, LPS upregulated the mRNA expression of the pro-inflammatory C-X-C motif chemokine ligand 8 (*CXCL8*) with little effect on interleukin 1 beta (*IL1B*), interleukin 6 (*IL6*), tumor necrosis factor alpha (*TNF-α*) and C-C motif chemokine ligand 2 (*CCL2*), whereas 3-HB treatment significantly reduced its expression (Figure 6b). CXCL8 is a well-established p38 MAPK-dependent inflammatory target in human intestinal epithelial cells, in which pharmacological p38 inhibition suppresses both CXCL8 transcript and protein induction by LPS and pro-inflammatory cytokines [45,46]. Together, these results indicate that 3-HB attenuates p38 MAPK activation and downstream *CXCL8* expression in HCT-8 cells, supporting its anti-inflammatory activity in human colonic epithelial cells at low micromolar concentrations. Thus, the cell-based assay extends the study beyond metabolite production and distribution by showing that 3-HB can directly modulate a host-relevant epithelial signaling pathway. This finding provides a functional link between engineered metabolite delivery and host-cell activity. Importantly, this cell-based evidence helps distinguish the specific contribution of 3-HB from the intrinsic effects of the probiotic yeast *S. boulardii*, suggesting that the biosynthesized 3-HB may exert a targeted anti-inflammatory effect. However, p38 MAPK activation in intestinal tissues and circulating inflammatory cytokines were not evaluated in SbDY02-treated mice, limiting our ability to directly correlate the cell-based findings with the *in vivo* effects of SbDY02. Additionally, because the *in vitro* anti-inflammatory effects in this study were evaluated primarily at the transcriptional level, the lack of direct measurements of secreted cytokines in the culture supernatant remains a limitation. To address these limitations, future studies should verify p38 MAPK modulation and inflammatory responses *in vivo* and directly quantify secreted cytokines *in vitro*.

## 4. Conclusions

In this study, we engineered the probiotic yeast *S. boulardii* to produce 3-HB by combining a heterologous biosynthetic pathway with rewiring of endogenous acetyl-CoA and ethanol metabolism. The optimized strain, SbDY02, produced 1.7 g/L 3-HB and increased intestinal 3-HB availability following oral administration in mice. SbDY02 treatment was also associated with altered fecal and circulating SCFA profiles, including increased fecal acetate and circulating butyrate and propionate. In HCT-8 cells, 3-HB also attenuated LPS-induced p38 MAPK activation and reduced *CXCL8* expression, supporting its anti-inflammatory activity at the epithelial-cell level. However, the lack of microbiome analysis, comprehensive biosafety assessment, and direct *in vivo* validation of inflammatory responses remain limitations that should be addressed in future studies. To our knowledge, this study provides the first demonstration of a 3-HB-producing probiotic yeast and supports engineered *S. boulardii* as a potential platform for localized intestinal metabolite delivery.

## Figures and Tables

**Table 1:** Probiotic yeast *Saccharomyces boulardii* strains and plasmids used in this study.

Strains/Plasmids	Description/Relevant Genotype ^1^	Reference
***Saccharomyces boulardii***		
WT Sb	Wild-type; *Saccharomyces boulardii* ATCC MYA−797	ATCC
SbDY01	WT Sb *int#11::PGK1*_P_*-ACS1-ADH7*_T_*-CCW12*_P_*-RephaB-CYC1*_T_*-TDH3*_P_*-RephaA-ADH4*_T_*-FBA1*_P_*-EctesB-MDH1*_T_	This study
SbDY01 *adh1*Δ	WT Sb *int#11::PGK1*_P_*-ACS1-ADH7*_T_*-CCW12*_P_*-RephaB-CYC1*_T_*-TDH3*_P_*-RephaA-ADH4*_T_*-FBA1*_P_*-EctesB-MDH1*_T_ *adh1*Δ	This study
SbDY01 *adh1*Δ *adh2*Δ	WT Sb *int#11::PGK1*_P_*-ACS1-ADH7*_T_*-CCW12*_P_*-RephaB-CYC1*_T_*-TDH3*_P_*-RephaA-ADH4*_T_*-FBA1*_P_*-EctesB-MDH1*_T_ *adh1*Δ *adh2*Δ	This study
SbDY01 *adh1*Δ *adh5*Δ	WT Sb *int#11::PGK1*_P_*-ACS1-ADH7*_T_*-CCW12*_P_*-RephaB-CYC1*_T_*-TDH3*_P_*-RephaA-ADH4*_T_*-FBA1*_P_*-EctesB-MDH1*_T_ *adh1*Δ *adh5*Δ	This study
SbDY01 *adh1*Δ *ADH7*	SbDY02; WT Sb *int#11::PGK1*_P_*-ACS1-ADH7*_T_*-CCW12*_P_*-RephaB-CYC1*_T_*-TDH3*_P_*-RephaA-ADH4*_T_*-FBA1*_P_*-EctesB-MDH1*_T_ *adh1*Δ *CCW12*_P_*-ADH7*	This study
**Plasmids**		
pRS41K-Cas9	pRS41K, a *KanMX* marker, Cas9	[24]
pRS42H_cg#1	pRS42H, a *hph* marker, gRNA block created 20-bp sequence (5′-GTACACCTACCCGTCACCGG)	[24]
pRS42H_INT#11	pRS42H, a *hph* marker, gRNA block for intergenic site#11	[25]
pRS42H_ADH1	pRS42H, a *hph* marker, gRNA block for deletion of *ADH1*	This study
pRS42H_ADH2	pRS42H, a *hph* marker, gRNA block for deletion of *ADH2*	This study
pRS42H_ADH5	pRS42H, a *hph* marker, gRNA block for deletion of *ADH5*	This study
pRS42H_ADH7	pRS42H, a *hph* marker, gRNA block for replacement of *ADH7*_P_	This study

^1^ The *tesB* gene derived from *Escherichia coli* TOP10 (*EctesB*, thioesterase), and two genes (*RephaA*, acetyl-CoA acetyltransferase; *RephaB*, acetoacetyl-CoA reductase) derived from *Ralstonia eutropha* H16 were codon-optimized for *Saccharomyces cerevisiae*.

## Data Availability

The original contributions presented in this study are included in the article/Supplementary Material. Further inquiries can be directed to the corresponding authors.

## Supplementary Materials

The following supporting information can be downloaded at: https://www.mdpi.com/article/10.3390/foods15173006/s1, Table S1: Primers and guide RNA sequences used in this study, Table S2: Heterologous gene sequences used for 3-HB production in this study, Table S3. Primer sequences used for qPCR analysis.

## Abbreviations

The following abbreviations are used in this manuscript:

3-HB	3-Hydroxybutyric acid
SCFA	Short-chain fatty acid
MAPK	Mitogen-activated protein kinase
*RephaA*	Acetyl-CoA acetyltransferase gene from *Ralstonia eutropha* H16
*RephaB*	Acetoacetyl-CoA reductase gene from *Ralstonia eutropha* H16
*EctesB*	Thioesterase gene from *Escherichia coli*
*GPD1*	Glycerol-3-phosphate dehydrogenase gene
*ALD6*	Aldehyde dehydrogenase gene
*ACS1*	Acetyl-CoA synthetase gene
*ADH*	Alcohol dehydrogenase gene
INT#11	Intergenic region #11
YP	Yeast extract-peptone
YPD20	YP medium supplemented with 20 g/L glucose
YPD40	YP medium supplemented with 40 g/L glucose
OD_600_	Optical density at 600 nm
sgRNA	Single-guide RNA
LBA	Luria–Bertani medium supplemented with 100 μg/mL ampicillin
DCW	Dry cell weight
HPLC	High-performance liquid chromatography
RI	Refractive index
PBS	Phosphate buffered saline
WT Sb	Wild-type yeast *Saccharomyces boulardii* MYA-797
LC	Liquid chromatography
MS	Mass spectrometer
MRM	Multiple reaction monitoring
ESI	Electrospray ionization
EMV	Electron multiplier voltage
RPMI	Roswell park memorial institute
FBS	Fetal bovine serum
