# Supplementary material for: Metabolic Engineering of Probiotic Saccharomyces boulardii Enables Intestinal 3-Hydroxybutyrate Delivery and Alters Short-Chain Fatty Acid Profiles in Mice
Source: Foods. 2026 Aug 26;15(17):3006. doi: 10.3390/foods15173006 (PMC13564623; doi:10.3390/foods15173006)
Supplement: Supplementary file 1 [file foods-15-03006-s001.zip › foods-4486147-supplementary.pdf]

foods

MDPI

Supplementary Material

Metabolic Engineering of Probiotic *Saccharomyces boulardii* Enables Intestinal 3-Hydroxybutyrate Delivery and Alters Short-Chain Fatty Acid Profiles in Mice

Deokyeol Jeong [1,2,3,†], Tongkewn Yoo [2,3,†], Jieun Woo [2,4], Luping Xu [2,3], Soo Rin Kim [5], Weicang Wang [2], Kee-Hong Kim [2,*] and Eun Joong Oh [2,3,*]

[1] Department of Food Science and Technology, Kongju National University, Yesan 32439, Republic of Korea; dyj@kongju.ac.kr
[2] Department of Food Science, Purdue University, West Lafayette, IN 47907, USA; yoo192@purdue.edu (T.Y.); woo89@purdue.edu (J.W.); xu653@purdue.edu (L.X.); wang6205@purdue.edu (W.W.)
[3] Whistler Center for Carbohydrate Research, Purdue University, West Lafayette, IN 47907, USA
[4] Interdepartmental Nutrition Graduate Program, Purdue University, West Lafayette, IN 47907, USA
[5] School of Food Science and Biotechnology, Kyungpook National University, Daegu 41566, Republic of Korea; soorinkim@knu.ac.kr
* Correspondence: kim618@purdue.edu (K.-H.K.); ejoh@purdue.edu (E.J.O.)
† These authors contributed equally to this work.

Table S1. Primers and guide RNA sequences used in this study.

Primers	Sequences (5′-) [1]	Description
OH706	TTATTCAGATCCATCGGGTTTTAGAGCTAGAAATAGCAAG	Insert_*SbADH1*_F
OH707	CGATGGATCTGAATAATTCGATCATTTATCTTTCACTGCG	Insert_*SbADH1*_R
OH983	AGGAGAGCATAGAAATGGTTTTAGAGCTAGAAATAGCAAG	Insert_*SbADH2*_F
OH984	ATTTCTATGCTCTCCTCTGGATCATTTATCTTTCACTGCG	Insert_*SbADH2*_R
OH1003	CTATCGAGGCTTCTACGGTTTTAGAGCTAGAAATAGCAAG	Insert_*SbADH5*_F
OH1004	GTAGAAGCCTCGATAGCTGGATCATTTATCTTTCACTGCG	Insert_*SbADH5*_R
OH1009	CAATTATCAGTTAAGCAGTTTTAGAGCTAGAAATAGCAAG	Insert_*SbADH7*_F
OH1010	GCTTAACTGATAATTGAATGATCATTTATCTTTCACTGCG	Insert_*SbADH7*_R
OH707	CGATGGATCTGAATAATTCGATCATTTATCTTTCACTGCG	Insert_*SbADH1*_R
OH983	AGGAGAGCATAGAAATGGTTTTAGAGCTAGAAATAGCAAG	Insert_*SbADH2*_F
OH500	GTAATTATCTACTTTTTACAACAAATATAAAACAAAAAATGTCGCCCTCTGCC	Donor_*ACS1*_F
OH501	TTAAGAGATTCTGAAAAATATTACGTATATAGATTATTACAACTTGACCGAATCAATTAG	Donor_*ACS1*_R
OH502	TTCGAATAAACACACATAAACAAACAAAAAAAATGACTGATGTTGTTATTGTTTC	Donor_*RephaA*_F
OH503	CGCATAATTGACGTTTATGAGTTCGTTCGATTTTTTTATTATTTCCTTTCCACCGCC	Donor_*RephaA*_R
OH504	GTCATTCGCTTAAACACTATATCAATAAAAAATGACTCAAAGAATTGCTTATG	Donor_*RephbB*_F
OH505	GTGAATGTAAGCGTGACATAACTAATTACATGATTATTAACCCATATGCAAACCAC	Donor_*RephbB*_R
OH506	ATAACCATAACCAAGTAATACATATTCAAAAAAAATGAGTCAGGCGCTAAAAAATTTA	Donor_*EctesB*_F
OH507	TTTTTCACTCTATTTCTGATCTTGAACAATTTATTAATTGTGATTACGCATCACCC	Donor_*EctesB*_R
OH508	TCCACTCCCCCATTTTTATCCGGATCTCTGCACGCAAAAGAAAACCTTCG	Donor_INT#11u_*CCW12*P_F
OH456	TGGGCTCAAGGTGACAAGGTCCTCGAAAATAGGGCCGCAAATTAAAGCCT	Donor_*TDH3*P_*CYC1*T_R
OH451	TCCACTCCCCCATTTTTATCCGGATCTCTGGTGAGTAAGGAAAGAGTGAG	Donor_INT#11u_*PGK1*P_F
OH509	GGGCGAAGTGCGCAACCTCGAAGGTTTTCTTTTGCGTGAATGTAGACGTACTCGACATG	Donor_*CCW12*P_*ADH7*T_R
OH510	GGGTTTTACCCATTCATGTCGAGTACGTCTACATTCACGCAAAAGAAAACCTTCG	Donor_*ADH7*T_*CCW12*P_F
OH456	TGGGCTCAAGGTGACAAGGTCCTCGAAAATAGGGCCGCAAATTAAAGCCT	Donor_*TDH3*P_*CYC1*T_R
OH457	TGGGACGCTCGAAGGCTTTAATTTGCGGCCCTATTTTCGAGGACCTTGTC	Donor_*CYC1*T_*TDH3*P_F
OH458	TTTACAGAAGTTGGAAGGCTGGTATTGTTGTTCAACTATTTTTCAGTCTTAAATGCTTC	Donor_*FBA1*P_*ADH4*T_R

OH459	CATACTGTTGGCTTTGAAGCATTTAAGACTGAAAAATAGTTGAACAACAATACCAGCC	Donor_*ADH4*$_{T}$_*FBA1*$_{P}$_F
OH460	CTGGCAAACGGCGCTCTCCAAGGCACAAACTTGTTTATTCATCATTATCATCATC	Donor_INT#11d_*MDH1*$_{T}$_R
OH710	GCACAATATTTCAAGCTATACCAAGCATACAATCAACTATCTCATATACACAAGTCGTC	Donor_*SbADH1*_F
OH711	GCTCTGTTACCGACGTAAGAACCAACAATAGAGATGGACTTGACGACTTGTGTATATGAG	Donor_*SbADH1*_R
OH987	CTCCAACTTATAAGTTGGAGAAATAAGAGAATTTCAGATTGAGAGAATGGCGGATCTC	Donor_*SbADH2*_F
OH988	GGCATGCTTGATAATGAAAACTATAAATCGTAAAGACATAAGAGATCCGCCATTCTCTC	Donor_*SbADH2*_R
OH1005	GATACCTAAAAAAGTTATTTAACTACATATCAACAAAATCAAAGCAACGTAACGAATTTG	Donor_*SbADH5*_F
OH1006	CAAAATAGCTTATATAAAAAGTAAAAATATATTCATCAAATTCGTTACGTTGCTTTG	Donor_*SbADH5*_R
OH1011	GTTGCAATTTTCTCAGGGGTGGAGTGGAAATGGTTTGCACGCAAAAGAAAACCTTCG	Donor_*SbADH7*_F
OH1012	CCGATGCCCTGAAATTTTTCTGGGTAAAGCATTTTTTATTGATATAGTGTTTAAGCGAAT	Donor_*SbADH7*_R
OH199	AGGAGTGAGAATGTGCGTTAGC	Conf_INT#11_F
OH201	GGCAATGCAAATTCTTCCAGAC	Conf_INT#11_R
OH712	CGGCCTTCCTTCCAGTTACTTG	Conf_*SbADH1*_F
OH713	ACCTGAGAAAGCAACCTGACC	Conf_*SbADH1*_R
OH989	GCGGGAAACCATCCACTTCAC	Conf_*SbADH2*_F
OH990	AGGGATGGTGTTTACTCGTGC	Conf_*SbADH2*_R
OH1007	CGGGATCGTTCCTTCGTATTGTC	Conf_*SbADH5*_F
OH1008	TCTCGCAAAGTACAGAATCACTCG	Conf_*SbADH5*_R
OH1013	GTAGATCAGGGACTATGCGAGC	Conf_*SbADH7*_F
OH1014	TGACTACCGAAGAATTGGTTTAAGG	Conf_*SbADH7*_R
**gRNA**	**guide RNA and PAM sequences (5′-)**	**References**
cg#1	GTACACCTACCCGTCACCGG AGG	[42]
INT#11	TGTGACAAAATAGAATCCAG CGG	[25]
SbADH1	GAATTATTCAGATCCATCGG TGG	This study
SbADH2	CAGAGGAGAGCATAGAAATG GGG	This study
SbADH5	CAGCTATCGAGGCTTCTACG AGG	This study
SbADH7$_{P}$	ATTCAATTATCAGTTAAGCA AGG	This study

[1] The flanking region is underlined.

Table S2. Heterologous gene sequences used for 3-HB production in this study.

Gene name (Accession number)	Sequences (5′-)	Codon optimization
RephaA (PZ713508)	ATGACTGATGTTGTTATTGTTTCTGCTGCTAGAACTGCTGTTGGTAAATTTGGTGGTTCTTTGGCTAAAATTCCTGCTCCTGA<br>ATTGGGTGCTGTTGTTATTAAAGCTGCTTTGGAAAGAGCTGGTGTTAAACCTGAACAAGTTTCTGAAGTTATTATGGGTCA<br>AGTTTTGACTGCTGGTTCTGGTCAAAATCCTGCTAGACAAGCTGCTATTAAAGCTGGTTTGCCTGCTATGGTTCCTGCTATG<br>ACTATTAATAAAGTTTGTGGTTCTGGTTTAAAGGCTGTGATGTTGGCTGCAAACGCTATTATGGCTGGTGATGCTGAAATT<br>GTTGTTGCTGGTGGTCAAGAAAATATGTCTGCTGCTCCACATGTTTTGCCTGGTTCTAGAGATGGTTTTAGAATGGGTGATG<br>CTAAATTGGTTGATACTATGATTGTTGATGGTTTGTGGGATGTTTATAATCAATATCATATGGGTATTACTGCTGAAAATGT<br>TGCTAAAGAATATGGTATTACTAGAGAAGCTCAAGATGAATTTGCTGTTGGTTCTCAAAATAAAGCTGAAGCTGCTCAAA<br>AAGCTGGTAAATTTGATGAAGAAATTGTTCCTGTTTTGATTCCACAAAGAAAAGGTGATCCTGTTGCTTTTAAAACTGATG<br>AATTTGTTAGACAAGGTGCTACTTTGGATTCTATGTCTGGGCTAAAACCTGCTTTTGATAAAGCTGGTACTGTTACTGCCGC<br>TAACGCCTCTGGTTTGAATGATGGTGCTGCTGCTGTTGTTGTTATGAGCGCTGCTAAAGCTAAAGAATTGGGTTTGACTCC<br>ATTGGCTACTATTAAATCTTATGCTAATGCTGGTGTTGATCCAAAAGTTATGGGTATGGGTCCTGTTCCTGCTTCTAAAAGA<br>GCTTTGTCTAGAGCTGAATGGACTCCACAAGATTTGGATTTGATGGAAATTAATGAAGCTTTTGCTGCTCAAGCTTTGGCT<br>GTTCATCAGCAAATGGGTTGGGATACTTCTAAAGTTAATGTTAATGGTGGTGCTATTGCTATTGGTCATCCAATTGGTGCTT<br>CTGGTTGTAGAATTTTGGTTACTTTGTTGCATGAAATGAAAAGAAGAGATGCTAAAAAAGGTTTGGCTTCTTTGTGTATTG<br>GTGGTGGTATGGGTGTTGCTTTGGCGGTGGAAAGGAAATAA	Yes
RephaB (PZ713509)	ATGACTCAAAGAATTGCTTATGTTACTGGTGGTATGGGTGGTATTGGTACTGCTATTTGTCAAAGATTGGCTAAAGATGGT<br>TTTAGAGTTGTTGCTGGTTGTGGTCCAAATTCTCCAAGAAGAGAAAAATGGTTGGAACAACAAAAAGCTTTGGGTTTTGA<br>TTTTATTGCTTCTGAAGGTAATGTTGCGGATTGGGATTCTACTAAAACTGCTTTTGATAAAGTTAAATCTGAAGTGGGTGA<br>AGTTGATGTTTTGATTAATAATGCTGGCATTACGAGAGATGTTGTTTTTAGAAAAATGACTAGAGCAGACTGGGATGCTGT<br>TATTGATACAAACTTGACTTCTCTTTTTAACGTAACTAAACAAGTTATTGATGGTATGGCTGATAGAGGTTGGGGTAGAAT<br>TGTTAATATTTCTTCTGTTAATGGTCAAAAAGGTCAATTTGGTCAAACTAATTATTCTACTGCTAAAGCTGGTTTGCATGGT<br>TTTACTATGGCTTTGGCTCAAGAAGTTGCTACTAAAGGTGTTACTGTTAATACTGTTTCTCCTGGTTATATTGCTACTGATAT<br>GGTTAAAGCTATTAGACAAGATGTTTTGGATAAAATTGTTGCTACTATTCCTGTTAAAAGATTGGGTTTGCCTGAAGAAAT<br>TGCTTCTATTTGTGCTTGGTTATCTTCTGAAGAATCTGGTTTTTCTACTGGTGCTGATTTTTCTTTGAATGGTGGTTTGCATAT<br>GGGTTAA	Yes
EctesB (CP184745.1)	ATGAGTCAGGCGCTAAAAAATTTACTGACATTGTTAAATCTGGAAAAAATTGAGGAAGGACTCTTTCGCGGCCAGAGTG<br>AAGATTTAGGTTTACGCCAGGTGTTTGGCGGCCAGGTCGTGGGTCAGGCCTTGTATGCTGCAAAAGAGACCGTCCCTGAA<br>GAGCGGCTGGTACATTCGTTTCACAGCTACTTTCTTCGCCCTGGCGATAGTAAGAAGCCGATTATTTATGATGTCGAAACG<br>CTGCGTGACGGTAACAGCTTCAGCGCCCGCCGGGTTGCTGCTATTCAAAACGGCAAACCGATTTTTATATGACTGCCTCT<br>TTCCAGGCACCAGAAGCGGGTTTCGAACATCAAAAAACAATGCCGTCCGCGCCAGCGCCTGATGGCCTCCCTTCGGAAA	No

	CGCAAATCGCCCAATCGCTGGCGCACCTGCTGCCGCCAGTGCTGAAAGATAAATTCATCTGCGATCGTCCGCTGGAAGTC<br>CGTCCGGTGGAGTTTCATAACCCACTGAAAGGTCACGTCGCAGAACCACATCGTCAGGTGTGGATCCGCGCAAATGGTA<br>GCGTGCCGGATGACCTGCGCGTTCATCAGTATCTGCTCGGTTACGCTTCTGATCTTAACTTCCTGCCGGTAGCTCTACAGC<br>CGCACGGCATCGGTTTTCTCGAACCGGGGATTCAGATTGCCACCATTGACCATTCCATGTGGTTCCATCGCCCGTTTAATT<br>TGAATGAATGGCTGCTGTATAGCGTGGAGAGCACCTCGGCGTCCAGCGCACGTGGCTTTGTGCGCGGTGAGTTTTATACC<br>CAAGACGGCGTACTGGTTGCCTCGACCGTTCAGGAAGGGGTGATGCGTAATCACAATTAA

Table S3. Primer sequences used for qPCR analysis.

Gene	Sequences (5′-)	Description
GAPDH	ACAACTTTGGTATCGTGGAAGG	qPCR_GAPDH_F
	GCCATCACGCCACAGTTTC	qPCR_GAPDH_R
IL-1B	AGCTACGAATCTCCGACCAC	qPCR_IL-1B_F
	CGTTATCCCATGTGTCGAAGAA	qPCR_IL-1B_R
IL-6	CCTGAACCTTCCAAAGATGGC	qPCR_IL-6_F
	TTCACCAGGCAAGTCTCCTCA	qPCR_IL-6_R
CXCL8	ACTGAGAGTGATTGAGAGTGGAC	qPCR_CXCL8_F
	AACCCTCTGCACCCAGTTTTC	qPCR_CXCL8_R
TNF	GAGGCCAAGCCCTGGTATG	qPCR_ TNF_F
	CGGGCCGATTGATCTCAGC	qPCR_ TNF_R
CCL-2	CAGCCAGATGCAATCAATGCC	qPCR_CCL2_F
	TGGAATCCTGAACCCACTTCT	qPCR_CCL2_R

https://doi.org/10.3390/foods15173006
